# A comparative study of anxiety and depression in patients with bronchial asthma, chronic obstructive pulmonary disease and tuberculosis in a general hospital of chest diseases

**DOI:** 10.1186/1744-859X-7-7

**Published:** 2008-05-21

**Authors:** Georgios Moussas, Athanasios Tselebis, Athanasios Karkanias, Dimitra Stamouli, Ioannis Ilias, Dionisios Bratis, Kalliopi Vassila-Demi

**Affiliations:** 1 Attikon General Hospital, Second Psychiatric Department, Medical School, University of Athens, Greece; 2 Sotiria General Hospital of Chest Diseases, Psychiatric Department, Athens, Greece; 3 Elena Venizelou Hospital, Athens, Greece

## Abstract

**Background:**

Depression necessitating assistance from health professionals has a lifetime prevalence of 10%. Chronic disease increases comorbidity with mood and/or anxiety disorders. Patients with chronic pulmonary disease present with severely impaired functionality, chronic somatic and psychogenic pain, require frequent hospitalizations and have a dependency upon medical and nursing personnel. In the present study we assessed anxiety and depression in patients hospitalized for pulmonary disease in a pulmonary disease hospital.

**Methods:**

We assessed anxiety, using the Spielberger state-trait anxiety scale, and depression, using the Beck Depression Inventory, in 132 patients with pulmonary disease.

**Results:**

A total of 49.2% of the sample had moderate or severe depression and 26.5% had anxiety. Women had higher depression and anxiety scores than men (t test, p < 0.05). Depression was positively correlated with anxiety, age and time from diagnosis. Anxiety was positively correlated with depression and time from diagnosis (Pearson r = 0.62 and 0.29, p < 0.01). Patients with chronic obstructive pulmonary disease and bronchial asthma had higher depression scores than patients with tuberculosis (t test, p < 0.01).

**Conclusion:**

Depression and anxiety are very prevalent in patients with pulmonary disease, especially chronic disease. This may be a very important negative factor in patients' adaptation to the chronic course of their disease.

## Background

Among psychiatric diseases, depression, necessitating assistance from health professionals, has a lifetime prevalence of 10% [1]. Furthermore, in the general population, depression has a point prevalence of 2.3% to 4.9%. Up to 80% of patients with depression are either treated by non-mental health professionals or receive no treatment at all [2]. Chronic disease increases comorbidity with mood and/or anxiety disorders. Usually, the more serious the somatic disease is, the more probable it will be accompanied by mood and/or anxiety symptoms of variable severity. Failure to manage such mental health problems increases the patients' probability of suffering from complications, even lethal. The lifetime prevalence of mood disorder in patients with chronic disease is 8.9% to 12.9%, with a 6-month prevalence of 5.8% to 9.4%. [3,4].

According to findings from worldwide research, 20% of patients with somatic disease suffer from major depression [4]. In relevant studies in Greece, 28.1% of patients hospitalized in general medical or surgical hospital wards had depression [5,6].

In patients with pulmonary disease in particular, functionality may be severely impaired due to chronic psychogenic and somatic pain, frequent hospital admissions and dependency from medical and nursing personnel. The observed higher prevalence of depression and anxiety in patients with chronic pulmonary disease – compared to other chronic diseases – may be explained within this context. Despite the fact that clinical experience accepts high comorbidity in pulmonary patients, studies assessing and comparing anxiety and depression levels among patients with different pulmonary diseases are lacking in the Greek literature. In the present study we assessed anxiety and depressive symptoms in patients hospitalized in pulmonary clinics with bronchial asthma (BA), chronic obstructive pulmonary disease (COPD) or tuberculosis (TB).

## Methods

Depression was assessed with the Beck Depression Inventory (BDI), which is widely used, and has been standardized and used in the Greek population previously [7-9]. The BDI, one of the most popular depression rating scales, includes 21 items graded from 0 to 3. The inner coherence reliability is high and the re-test reliability ranges from 0.48 to 0.86 for clinical groups and 0.60 to 0.90 for non-clinical population. Its validity in relation to an external criterion for depression, such as clinical diagnosis, is considered to be satisfactory [7]. Anxiety was assessed with the Spielberger state-trait anxiety scale, one of the well-known and broadly used anxiety rating scales. The scale consists of 40 items, each one graded from 1 to 4. The scale differentiates anxiety to (a) anxiety caused by a specific condition (state subscale), and (b) anxiety as a more permanent characteristic of the personality (trait subscale). This second (trait) subscale was used in our study protocol. The scale is considered as having a high inner coherence reliability and validity compared to clinical diagnosis [10-12].

### Sample

The sample included 140 hospitalized patients, of which 8 subjects refused to participate and were therefore excluded. The study included 132 patients (78 men and 54 women) in the pulmonary departments of our hospital. Of them, 42 were diagnosed with BA, 60 with COPD and 30 with TB. They were considered for enrollment over a 2-month period. All the participants were informed and gave their formal consent.

The subjects replied to the questionnaires in the presence of psychologists and/or psychiatrists familiarized with such tests. We assessed age, gender, years of education, duration of illness and diagnosis for hospitalization. Student's t test was used to assess differences in anxiety or depression between genders and among BA, COPD or TB patients. Pearson's correlation was used to assess the impact of anxiety on depression (and vice versa) as well as the impact of age or time from diagnosis on anxiety or depression. Logistic regression was used to assess the presence of depression (i.e. BDI scores > 13) as a function of gender, age, time from diagnosis and anxiety. Descriptive statistics are given as mean ± standard deviation (SD).

## Results

The mean age of the sample was 54.08 ± 16.60 years and mean time from diagnosis was 8.78 ± 9.14 years. Men were older (57.44 ± 15.16 years) than women (49.22 ± 17.50 years, two-tailed t test, p < 0.05). There was no difference in the duration from diagnosis (two-tailed t test, p = 0.56). Women had higher anxiety and depression scores than men (two-tailed t test, p < 0.05); 49.2% of the sample had moderate to severe depression, and 44.0% of men had depression symptoms compared to 55.6% of women, whilst 21.8% of men had anxiety symptoms compared to 33.3% of women (Table 1).

**Table 1:** Patients studied by gender

**Gender**		**Age**	**Time from diagnosis (Years)**	**Anxiety**	**Depression**
Men (n = 78)	Mean	57.45	9.18	42.44	12.71
	SD	16.16	10.22	9.87	8.09
Women (n = 54)	Mean	49.22	8.30	46.20	15.50
	SD	17.05	7.76	10.13	8.14
Total (n = 132)	Mean	54.08	8.78	43.98	13.85
	SD	16.60	9.14	10.11	8.20

Depressive symptomatology was positively correlated with anxiety (Pearson r = 0.62, p < 0.01), age (Pearson r = 0.20, p < 0.05) and time from diagnosis (Pearson r = 0.39, p < 0.01). The correlation of depression and age persisted when time from diagnosis was used as a control variable (partial correlation two-tailed p < 0.05). Anxiety was positively correlated with duration of illness (Pearson r = 0.29, p < 0.01). When depression was used as a control variable, the correlation of anxiety with time from diagnosis was not maintained (partial correlation r = 0.12, p > 0.05). Patients with COPD had the higher depression scores, followed by patients with BA, whereas patients with TB had the lowest depression scores. Anxiety was higher in patients with COPD compared to patients with TB. Patients with COPD were older and had more years of illness compared to those with BA and TB (t test p < 0.05). BA patients were ill for a longer time compared to TB patients (t test p < 0.05) (Table 2).

**Table 2:** Patients studied by disease

**Gender**		**Age**	**Time from diagnosis (Years)**	**Anxiety**	**Depression**
Bronchial asthma (n = 42)	Mean	43.33	9.24	43.67	14.31
	SD	14.28	6.90	9.96	7.45
COPD (n = 60)	Mean	66.03	13.46	45.87	15.48
	SD	9.61	10.57	10.36	8.42
Tuberculosis (n = 30)	Mean	45.23	2.69	40.67	9.93
	SD	14.99	6.51	9.19	7.71
Total (n = 132)	Mean	54.08	8.78	43.98	13.85
	SD	16.60	9.15	10.11	8.20

COPD, chronic obstructive pulmonary disease.

We also examined depression in relation to anxiety, years of illness, gender and age by logistic regression. Logistic regression showed that anxiety seems to be the major determinant for depression (p < 0.001) (Table 3).

**Table 3:** Pearson correlation scores

		**Depression**	**Anxiety**	**Age**	**Time from diagnosis (Years)**
Depression	Pearson correlation coefficient (n = 132)	1.00	0.62	0.20	0.39
	Two-tailed p value	-	< 0.01	< 0.05	< 0.01
Anxiety	Pearson correlation coefficient (n = 132)	0.62	1.00	0.13	0.29
	Two-tailed p value	< 0.01	-	0.14	< 0.01
Age	Pearson correlation coefficient (n = 132)	0.20	0.13	1.00	0.34
	Two-tailed p value	< 0.05	0.14	-	< 0.01
Time from diagnosis	Pearson correlation coefficient (n = 107)	0.39	0.29	0.34	1.00
	Two-tailed p value	< 0.01	< 0.01	< 0.01	-

## Discussion

The present study confirms that COPD patients are the group with the higher and more severe depression comorbidity. Depression in patients with chronic respiratory diseases coexists with anxiety and is related to the chronicity of the disease in our study, which has a negative effect on quality of life [13].

In recent years there has been a growing interest in the relationship between chronic pain and depression [14]. Chronic respiratory diseases such as COPD and BA entail serious subjective difficulties, chronic psychogenic and somatic pain, frequent hospital admissions, hospital dependency and dependency on oxygen. This metaphorically and literally suffocating disease status may explain the high percentage of depression in patients with COPD and BA in the study, which was higher than the percentage reported in studies performed with in-patients of general hospitals [5]. Furthermore, this difference is verified by studies performed with patients with respiratory failure, with depression being observed in 30% of patients with moderate failure and in 50% in patients with severe obstructive pulmonary disease [15,16].

Anxiety and depression are very prevalent even in patients with moderate COPD (categorized as such by respiratory symptoms evaluation and functional tests using medical criteria) [17]. Chronic disease and comorbidity with anxiety and depression apparently leads to increased use of health services, approximately twice as often than in patients with no psychological burden [18]. Psychosocial stressors, such as death of a spouse or divorce, are closely related to relapses and aggravations of respiratory disease, especially in men, pointing to a link between psychological factors and chronic pulmonary disease [19]. Patients with COPD cannot cope adequately with everyday needs. This inadequacy may lead to heightened anxiety and depression, which in turn may worsen the everyday inadequacy. It has been reported that this is (probably) a factor that leads BA and COPD patients to frequent hospital admissions and even intensive care unit hospitalizations [20].

It is accepted that current psychiatric practice has valid ways to diagnose depression, implementing different diagnostic criteria and taxonomic systems such as the Diagnostic and Statistic Manual IV (DSM-IV) and International Classification of Disorders (ICD-10). The pathogenesis of depression is becoming better understood and therapy has a very high success rate. This progress sets the necessity for a more successful detection of all forms of depression, especially in chronic somatic patients and in the elderly; a group in which depression often escapes diagnosis, although in the elderly COPD is an important cause of morbidity, disability and mortality [21]. As mentioned previously, COPD and BA are chronic diseases with severe subjective difficulties, dependency on medical and nursing personnel and dependency on oxygen. In these diseases depression ranges from 30% in moderate up to 50% in severe forms of COPD [15], a fact also verified in our study. Depression may be a very important negative factor to treatment adherence for patients with somatic disease. Additionally, it may hinder adaptation to chronic disease conditions and it is known that adaptation is a crucial survival factor in chronic diseases [22].

## Conclusion

Patients suffering from BA and COPD have a significantly higher rate of anxiety and depression compared to the general population. A probable cause is the chronicity and severity of pulmonary disease. Detection and management of these mental disorders may ameliorate prognosis of the pulmonary disease and improve adaptation and quality of life of these patients.

## Competing interests

The authors declare that they have no competing interests.
